# Microstructure Evolution of Super304H Austenitic Steel During Long-Term Creep at 700 °C

**DOI:** 10.3390/ma18081756

**Published:** 2025-04-11

**Authors:** Jiale Zhang, Zhengfei Hu, Ziyi Gao

**Affiliations:** School of Materials Science and Engineering, Tongji University, Shanghai 202409, China; 2132861@tongji.edu.cn (J.Z.); 2032923@tongji.edu.cn (Z.G.)

**Keywords:** austenitic steel, microstructure evolution, precipitation strengthening, intergranular damage, EBSD

## Abstract

Creep tests of Super304H austenitic steel were carried out at 700 °C under different stresses. The samples were characterized by an optical microscope (OM), scanning electron microscope (SEM) and a transmission electron microscope (TEM). The results show that high-temperature creep promotes the precipitation of the M_23_C_6_, secondary MX carbide, σ phase, Cu-rich phase and Z phase. These fine precipitates improve both the matrix and grain boundary strength. Furthermore, the precipitation sequence of these second phases relates to the stress level during elevated temperature testing. The rapid precipitation of the σ phase is also observed at high stress levels, whereby fast growth at triangle boundaries notably deteriorates grain boundary strength. Conversely, the presence of dispersed fine MX precipitates under low-stress conditions during long-term creep should contribute significantly to microstructure stability and long-term creep strength. Despite the absence of homogeneous cavities observed on the grain boundary when subjected to creep for over 20,000 h, the decrease in grain boundary strength was explained from another aspect by analyzing the change in low angle grain boundary during creep.

## 1. Introduction

In recent years, ultra-supercritical (USC) power plants have continuously increased steam pressure and temperature to meet demands for energy conservation and environmental protection [1,2]. Super304H austenitic heat-resistant steel is widely used in USC units for superheater and reheater components due to its excellent resistance to corrosion and oxidation and creep strength at high temperatures [3,4]. Super304H austenitic heat-resistant steel is based on 18/8 Cr-Ni stainless steel, with additions of about 3% Cu and small amounts of Nb and N. N mainly plays a role in solid solution strengthening, while fine M_23_C_6_ carbides and Nb (C, N) and copper-rich phases dispersed in the matrix region provide precipitation strengthening [5,6]. Among these precipitates, the copper-rich phase is considered to be the most important strengthening phase in Super304H steel [7].

Long-term use in the temperature range of 600–700 °C causes the evolution of the microstructure, especially the precipitates, leading to a certain degree of degradation of the material. One of the most successful ways to improve the long-term creep resistance of austenitic steels is to increase the degree of precipitation strengthening during creep. This can be achieved by adding small amounts of strong carbide/nitride forming elements (such as niobium or nitrogen) to these steels or using heat treatment methods. C. L. Zhang et al. [8] found that controlling the content of niobium in steel can stabilize the supersaturated carbon element by forming Nb (C, N) to inhibit the formation of M_23_C_6_, thereby reducing the intergranular corrosion (IGC) sensitivity of Super304H steel. Moon, et al. [9] found that increasing the nitrogen content in the solid solution can enhance the matrix and effectively improve the high-temperature tensile strength of the steel. Chi et al. [10] found that copper-rich particles precipitate in Super304H steel after aging for 5 h, and Cu atoms gradually aggregate to Cu-rich particles as aging time increases. Even after long-term aging, Cu-rich particles still maintain their nanometer size and are evenly distributed within the grains. This is one of the key reasons Super304H heat-resistant steel retains good strength at high temperatures. Many studies have investigated the effect of temperature on the microstructure evolution of this steel during long-term use. Xiao Jin [11] et al. found that while the evolution of precipitates during aging can improve the tensile properties and hardness of the material, it also reduces the impact energy of the material. A. Zielińsk et al. [12] studied the evolution of precipitates in Super304H steel during aging.

Xue Wang et al. [13] studied the relationship between microstructure evolution and the mechanical properties of Super304H steel after high-temperature aging. Regarding creep behavior, more attention has been paid to microstructure characteristics, especially the evolution of precipitates during long-term creep at high temperatures [14]. During the creep process, the grain boundary strength may be damaged by the precipitation of intergranular carbides (mainly M_23_C_6_ rich in Fe (Cr)). Additionally, the σ phase, which more severely weakens the grain boundary performance, precipitates after a certain period of creep [15].

Hong et al. [16] studied the relationship between the formation of M_23_C_6_ precipitates at grain boundaries and the characteristics of grain boundaries in austenitic steels. They found that precipitation at highly random and poorly oriented grain boundaries is more harmful to the properties than the CSL interface. The CSL grain boundary is a kind of high-angle grain boundary (the orientation rotation angle θ value between the two grains is greater than 15°). The crystal lattice of two grains with the same crystal structure is infinitely extended to space, and one of the grains is allowed to rotate a specific angle around a low-index crystal axis, so that some of the lattice points in the two crystal lattices overlap regularly in space to form a new lattice. Using Σ to represent the coincidence degree of the coincidence position lattice, the smaller the Σ value is, the higher the CSL density is. Gaurav Singh et al. [17] strengthened the grain boundary by changing the morphology of M_23_C_6_. It was found that the volume fraction of Ʃ3 twin boundaries increased significantly after strengthening, which greatly hindered fatigue crack propagation behavior. Simultaneously, the pinning effect of M_23_C_6_ improved creep resistance. However, there are few studies on the evolution of the precipitated phase of Super304H during high-temperature creep, especially regarding the conditions of the Z phase (secondary NbCrN). Based on several previous studies, Kazuhiro Kimura [18] suggested that the Z phase transforms from Nb (C, N) during creep. Jae-Hyeok Shim [19] simulated Z phase precipitation after 1000–10,000 h of creep, predicting that its growth was relatively slow, with a maximum size of about 10 nm after 10,000 h.

Previously, the mechanical properties of Super304 austenitic steel at elevated temperatures were typically investigated during the aging process without additional stress loading. Some creep tests and investigations into microstructure evolution were conducted at 650 °C [20,21]. However, in the literature, there is generally still a lack of comprehensive information on the creep behavior and evolution of microstructure at more than 20,000 h at 700 °C. In this paper, the creep tests of Super304H steel at 700 °C were carried out at different stress levels. The creep rate of each stage of microstructure degradation and the precipitation sequence under creep conditions was determined, and the microstructure stability was well analyzed.

## 2. Experimental Procedures

### 2.1. Materials

**Figure 1:**
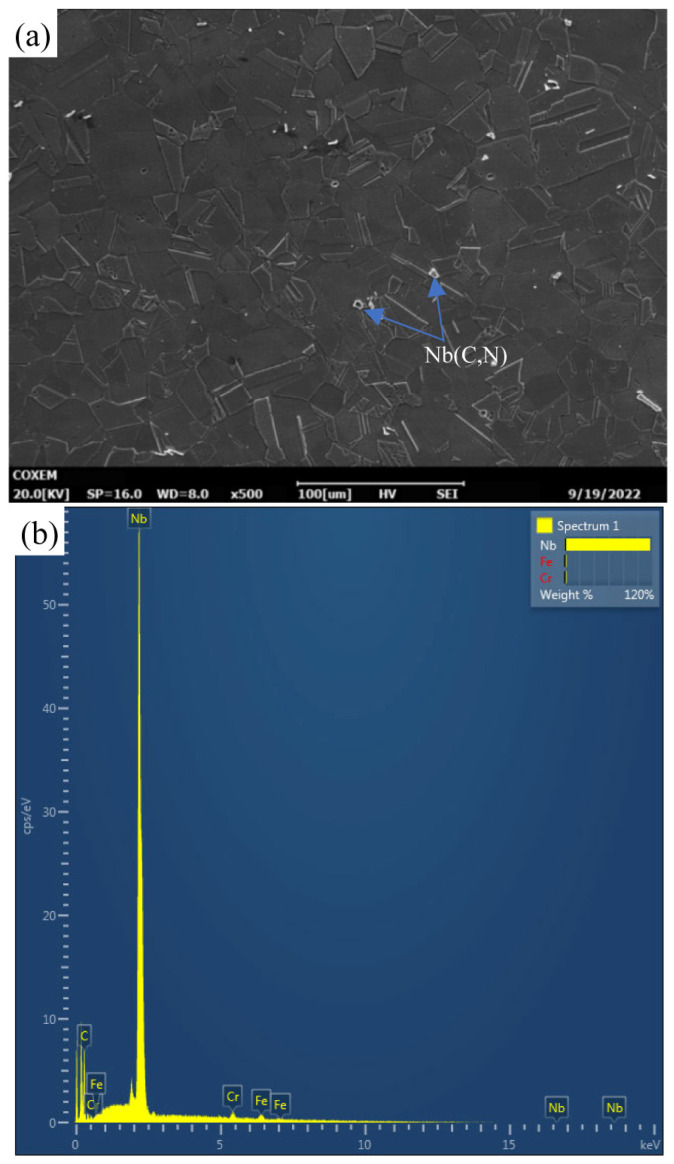
SEM image (**a**) and EDS pattern (**b**) of the as-received Super304H austenitic steel.

The samples were cut from commercial pipe products prepared using conventional processes, followed by final solid solution heat treatment at a solution temperature of 1100 °C. The chemical composition of Super304H tubes, meeting the ASTM standard [22] requirements, is detailed in Table 1. A scanning electron microscope (SEM) image of a Super304H tube in its as-received state is shown in Figure 1, revealing clear grain boundaries and many twin substructures within austenite grains. Energy dispersive X-ray spectroscopy (EDS) analysis showed that the content of niobium in the primary phase was relatively high. According to the results of previous studies [8,10,12], the primary phase should be the MX phase. The average grain size of the original sample measures about 20 μm.

### 2.2. Creep Tests

**Figure 2:**
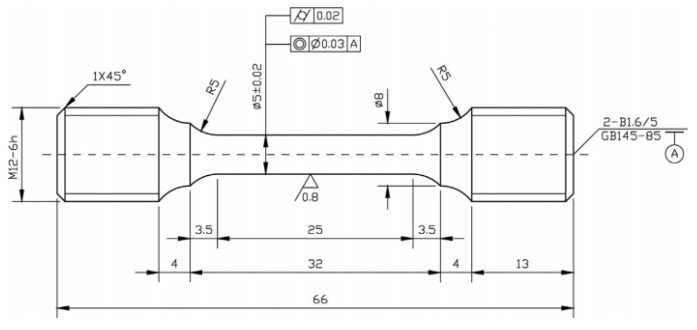
Dimensions of creep sample (unit: mm).

A series of standard creep rupture tests were carried out on Super304H austenitic heat-resistant steel specimens at 700 °C until fracture. The dimensions of the creep samples are displayed in Figure 2. The specimens were subjected to constant loads at stress levels of 80, 110, 150 and 180 MPa.

### 2.3. Microstructure Characterization

The observed samples were cut from the creep fracture specimens using wire electrical discharge machining. The specimens for metallographic and SEM observations were polished and etched in CuCl_2_ + HCl + H_2_O solution. Transmission electron microscope (TEM) samples were mechanically polished, double-jetted electrolytically and observed using a JEM-2100F transmission electron microscope(Japan Electronics Co., Ltd., Tokyo, Japan. For fine precipitation observation, TEM samples were prepared using the replica method. Electron backscatter diffraction (EBSD) analysis was performed using an EBSD system (Oxford C-nano) connected to a field emission scanning electron microscope (ZEISS, Baden-Württemberg, Germany). Prior to EBSD observation, the samples were mechanically polished and then electrolytically polished for 30 s at a voltage of 22 V with a current of 1.5–2.0 A in an electrolyte composed of 10% perchloric acid and 90% alcohol at 0 °C. The data obtained were processed and analyzed using Channel 5 2019 v5.12 software.

### 2.4. Microhardness Tests

The Vickers hardness of the polished samples was tested using an HVS-1000A digital microhardness tester (Jinan Fangyuan Test Instrument Co., Ltd., Jinan, China) under a constant load of 1.96 N and a holding time of 15 s.

## 3. Results

### 3.1. Creep Rupture Test Results and Microhardness

The results of creep rupture tests are listed in Table 2. It was determined that creep rupture time increases with the decrease in the stress levels of loads. The endurance time is 548 h under stress of 180 MPa, and it is up to 21,462 h under stress of 80 MPa.

**Figure 3:**
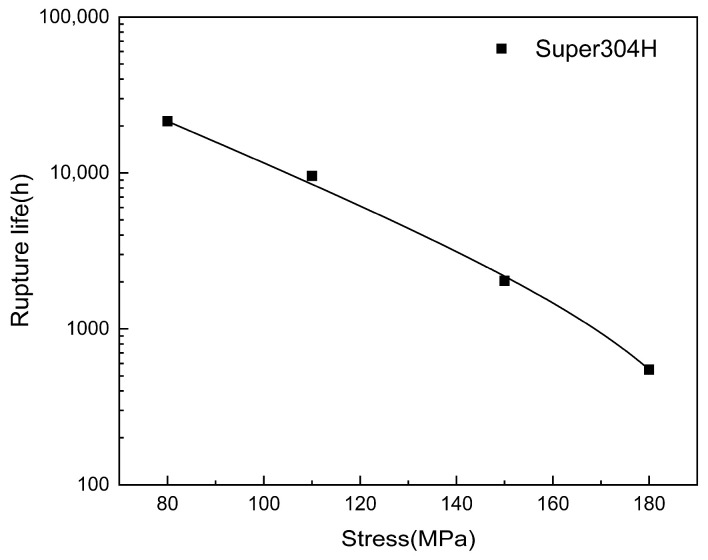
Variations in applied stress as a function of rupture life of Super304H steel at 700 °C.

Figure 3 shows the logarithmic relationship curve between the fracture life of Super304H (18Cr-9Ni-3Cu-Nb-N) steel and the applied stress. The curve shows that the long-term rupture strength of the steels can be expressed by the following polynomial fitting curve.

t = 236,545.4 − 237,128.7(1 − e^(−σ/33.7)^) (1)

where t is the creep rupture time (h), σ is the applied stress (MPa) and A and B are constants that relate to the material and test temperatures. Therefore, the fracture stress of the steel after creep at 700 °C for 10^5^ h can be inferred by the extrapolation method as follows,

(2) $$\mathrm{Super}304H:\;\sigma_{10^{5}}^{700}=62.01\mathrm{MPa}$$

According to these results, the properties of these materials meet the requirements of ASTM standards [22]. Theoretically, at 700 °C, the steel can guarantee safe service for 10^5^ h under stress below 60 MPa.

**Figure 4:**
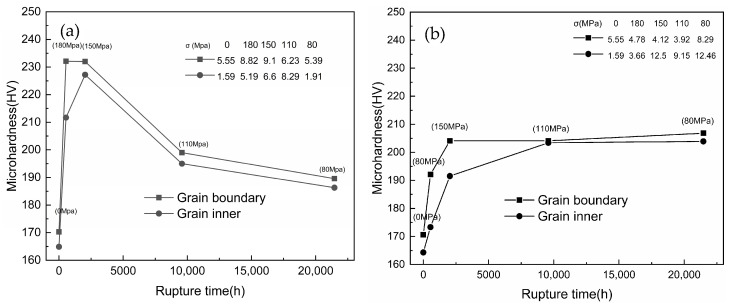
Microhardness change in creep fracture specimen at 700 °C: (**a**) the gauge portions and (**b**) the grip end (the σ in the table in the figure is the standard deviation).

Figure 4 shows the microhardness of the gauge portions taken from samples subjected to creep, along with the same samples from the grip end. It is clear the hardness decreased significantly with creep time, both in the matrix and at the grain boundary, and the hardness of the grain interior and grain boundary of the grip end increases first and then remains stable with the increase in creep time

### 3.2. Microstructure

#### 3.2.1. General Characteristics

As shown in Figure 1, the microstructure of the as-received Super304H steel consists of annealing twin austenite, with few primary precipitates of Nb (C, N) in the grains. These primary precipitates are believed to effectively inhibit grain growth during heat treatment in Super304H steel.

**Figure 5:**
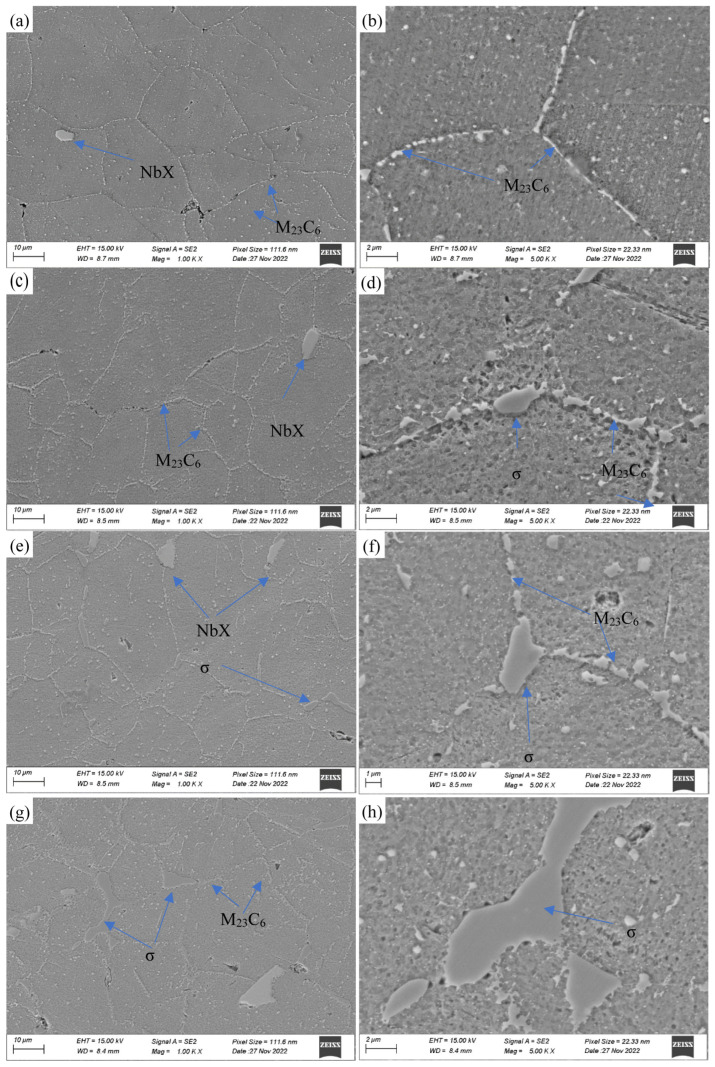
SEM images of the samples after creep rupture at 700 °C. (**a**,**b**) 180 MPa/548 h; (**c**,**d**) 150 MPa/2034 h; (**e**,**f**) 110 MPa/9584 h; (**g**,**h**) 80 MPa/21,462 h.

The presence of strong carbide-forming elements such as niobium or titanium also affects the precipitation of second phases during creep [23]. It can be seen from Figure 5 that the primary phase of Nb (C, N) exhibits high stability, as its size does not increase significantly even after creep durations exceeding 20,000 h at 700 °C.

The microstructures of the samples subjected to creep observed by the SEM are shown in Figure 5. As shown in Figure 5a,b, one notable feature in the samples subjected to creep is the presence of some small cracks or cavities formed along grain boundaries, particularly noticeable in samples subjected to high stress levels (180 and 150 MPa). However, fewer cavities formed along grain boundaries under low stress, even after creep times exceeding 20,000 h.

Another observed phenomenon is the formation of precipitates during the creep process. After creep for 548 h, carbides identified as M_23_C_6_, which are the most common precipitates in heat-resistant steel, were precipitated at the grain boundaries. The precipitates largest in size were generally precipitated at triangular boundaries and exhibit exponential growth in size with creep time. Both these precipitates and the very fine ones will be described as follows.

#### 3.2.2. Precipitates at Grain Boundary

**Figure 6:**
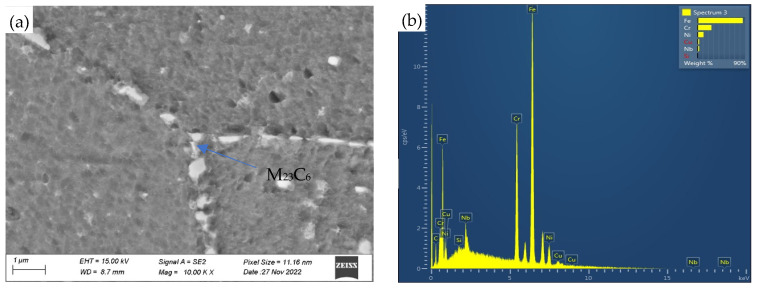
SEM images (**a**) and corresponding EDS energy spectrum (**b**) of carbides at grain boundary in 180 MPa/548 h sample.

**Figure 7:**
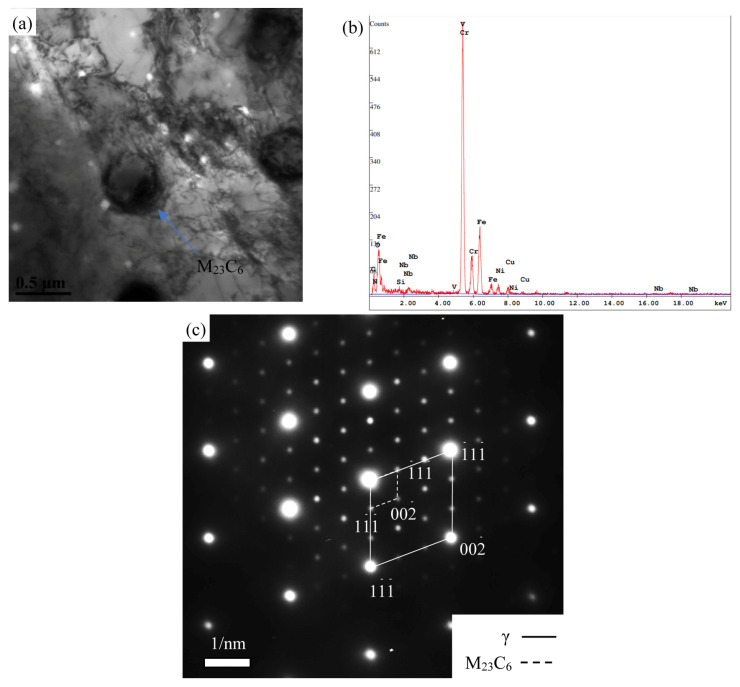
TEM images (**a**), corresponding EDS energy spectrum (**b**) and SAED (**c**) of M_23_C_6_ phase inside the grains in 180 MPa/548 h sample.

**Figure 8:**
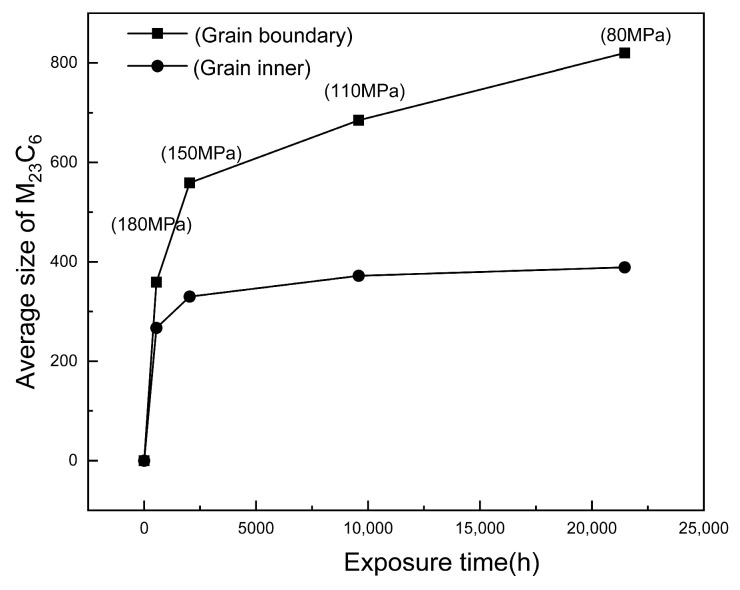
Size change of M_23_C_6_ precipitates in Super304H steel after creep at 700 °C (the σ in the table in the figure is the standard deviation).

After creep at 700 °C for 548 h, M_23_C_6_ and the primary MX phase were identified in the grain boundaries through the SEM with EDS analysis. As shown in Figure 6, the precipitate on the grain boundary is confirmed to be M_23_C_6_ due to its Cr-rich characteristics. In general, the fine M_23_C_6_ precipitates formed at the beginning of creep can effectively pin the grain boundary and strengthen the creep resistance. The average equivalent diameter of M_23_C_6_ particles was measured as the parameter for determining their size. In addition, the precipitation of M_23_C_6_ was observed inside the grains using diffraction and energy spectrum analysis, shown in Figure 7. The weighted average phase size of the measured M_23_C_6_ precipitates with the increase in creep time is plotted in Figure 8 for comparison. It is evident that as creep time increases, the size of M_23_C_6_ precipitates in both the grain boundary and the grain increases significantly, although the growth rate decreases markedly with creep time.

**Figure 9:**
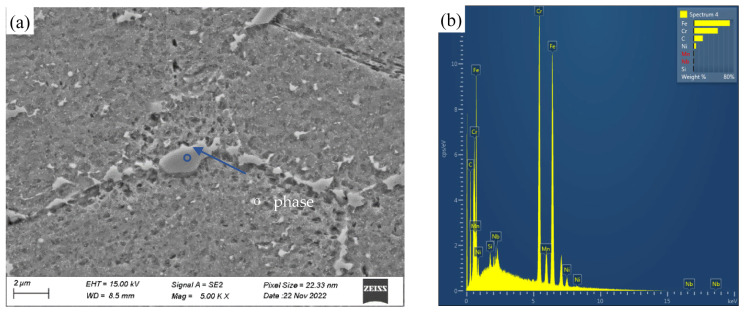
SEM images (**a**) and corresponding EDS energy spectrum (**b**) of the precipitated phase at grain boundary in 150 MPa/2034 h sample.

In general, the larger precipitated phase in the precipitated phase is the σ phase. The σ phase was first observed at the grain boundary of the 150 MPa/2034 h creep specimen. As shown in Figure 5, under the experimental conditions, the growth of the σ phase is very rapid with the increase in creep time. In addition, to avoid errors and distinguish M_23_C_6_, the sample was analyzed using EDS (Figure 9). The corresponding EDS results clearly indicate that it is an intermetallic compound phase containing Fe and Cr elements. Figure 5 clearly shows that the first precipitated phase is M_23_C_6_, followed by the σ phase. Since the nucleation and propagation of creep damage may occur at the carbide/matrix interface, these coarsening precipitates at the grain boundaries are considered detrimental to creep strength.

#### 3.2.3. Intragranular Precipitates

The TEM was employed to analyze the samples subjected to creep by observing the morphology and distribution of the fine precipitates and identifying them via diffraction composition. M_23_C_6_ also nucleated in the matrix, as shown in Figure 7. The statistical sizes of the M_23_C_6_ precipitates are shown in Figure 8 with the quantitative comparison indicating that the growth rate of the M_23_C_6_ precipitates at the grain boundary is slightly higher than that in the matrix. This difference is attributed to the diffusion mechanism during the nucleation process and the interface energy [24].

**Figure 10:**
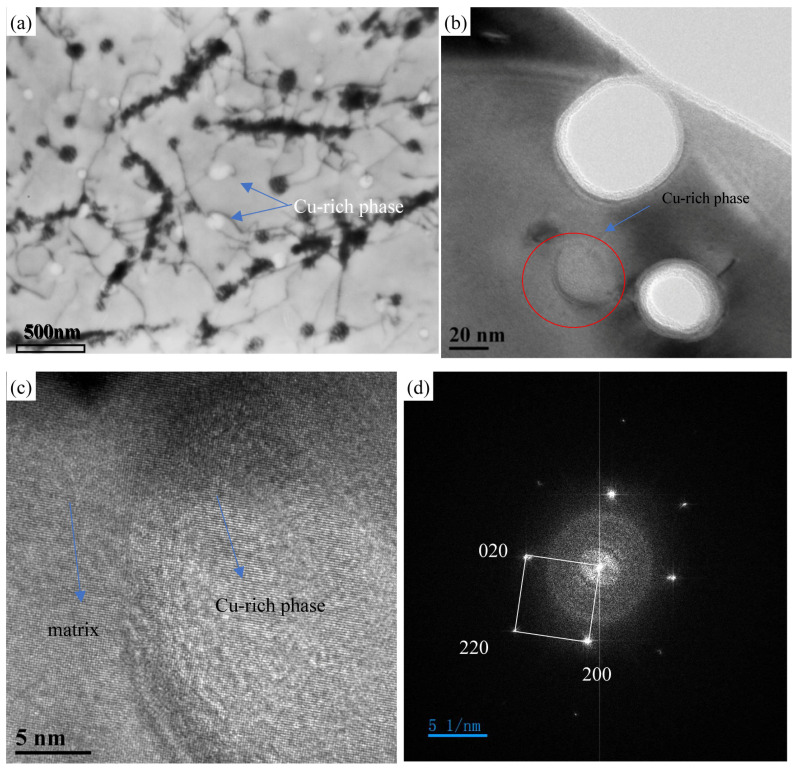
TEM images of precipitates for 180 MPa/548 h sample: (**a**,**b**) morphology of precipitates; (**c**), high-resolution image of Cu-rich phase; (**d**) Fourier transform of high-resolution images.

In addition to the M_23_C_6_ precipitates, a large number of Cu-rich phases precipitated in the matrix. It is noted that the copper-rich phase is the most important strengthening phase of Super304H, ready to precipitate at the beginning of creep [25,26], exhibiting high volume fraction, density and good stability with uniform distribution within the grains. The TEM images in Figure 10a show the dispersive Cu-rich phase precipitates along dislocations after 548 h of creep, proving the effective role of the Cu-rich phase in pining dislocations and slowing down their movement dislocations, thereby greatly improving the creep strength of Super304H. After creep at 700 °C for 548 h, Figure 10a,b reveal that there are dense and uniformly distributed Cu-rich phases that are nearly spherical in shape in Super304H steel. The finely dispersed Cu-rich phases inside the grains have considerable stability. As the creep time increases, the particle size of the Cu-rich phase continues to slowly increase, potentially ensuring that the steel maintains high strength over a long service life [27].

Figure 10c,d show the TEM images of Super304H steel after creep at 700 °C for 548 h, reflecting the relationship between the Cu-rich phase and the matrix. Figure 10c shows that the Cu-rich phase is coherent with the matrix. This proves that the Cu-rich phase has a similar crystal structure and lattice parameters compared to the matrix [28].

**Figure 11:**
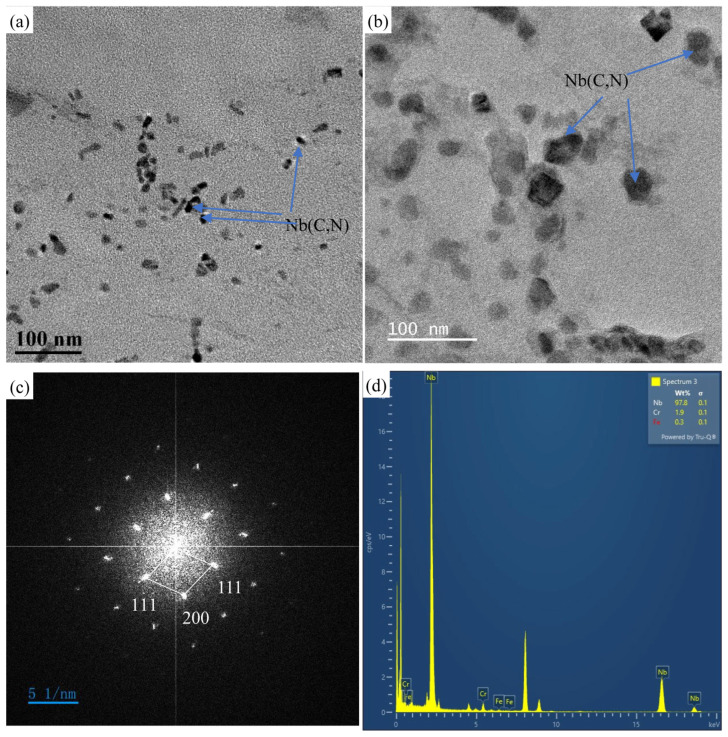
TEM images of the replica. (**a**) 180 MPa/548 h and (**b**) 80 MPa/21,462 h creep fracture specimens. (**c**) Fourier transform of high-resolution images, (**d**) EDS spectrum of Nb (C,N).

Figure 11 shows that after creep at 700 °C for 21,462 h, a large number of secondary MX precipitates (of nanoscale sizes) were observed in the Super304H steel sample. Secondary nano-MX precipitates can improve the mechanical properties and creep resistance of steel, and this strengthening effect is better than that of most traditional heat-resistant steels. Steel strengthened by secondary nano-MX precipitates can maintain stable high-temperature strength even after long-term aging [12,29,30]. Therefore, it is considered that the main strengthening phases of Super304H steel under high-temperature creep conditions are the Cu-rich phase and secondary MX precipitates. Although the Cu-rich phase is the main strengthening phase in the crystal, the strengthening effect of the secondary MX precipitated phase cannot be ignored under high-temperature conditions.

**Figure 12:**
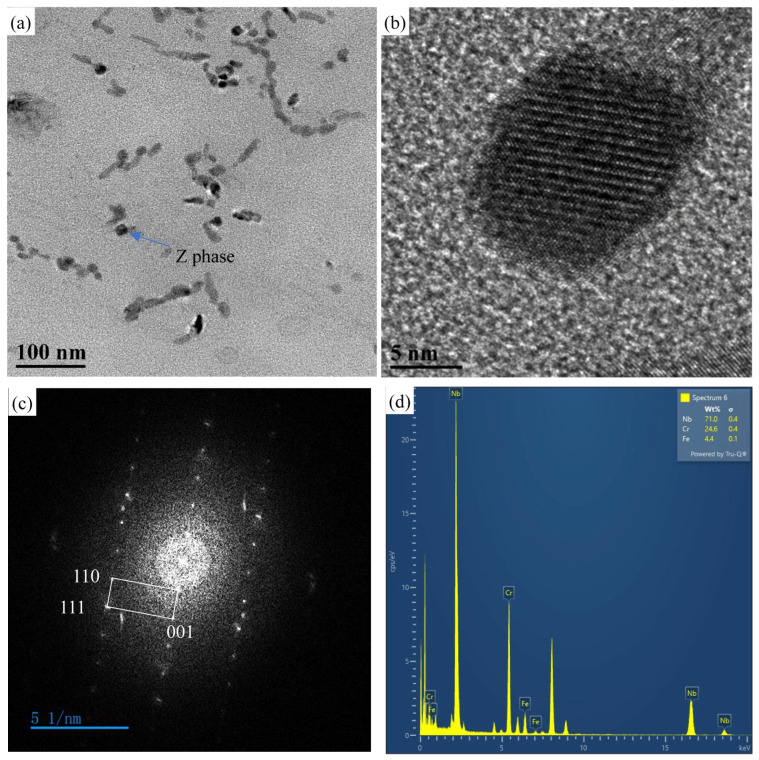
(**a**) TEM images of the replica of the 180 MPa/548 h creep fracture specimen, (**b**) high-resolution image of Z phase, (**c**) Fourier transform of high-resolution image, (**d**) corresponding EDS spectrum.

**Figure 13:**
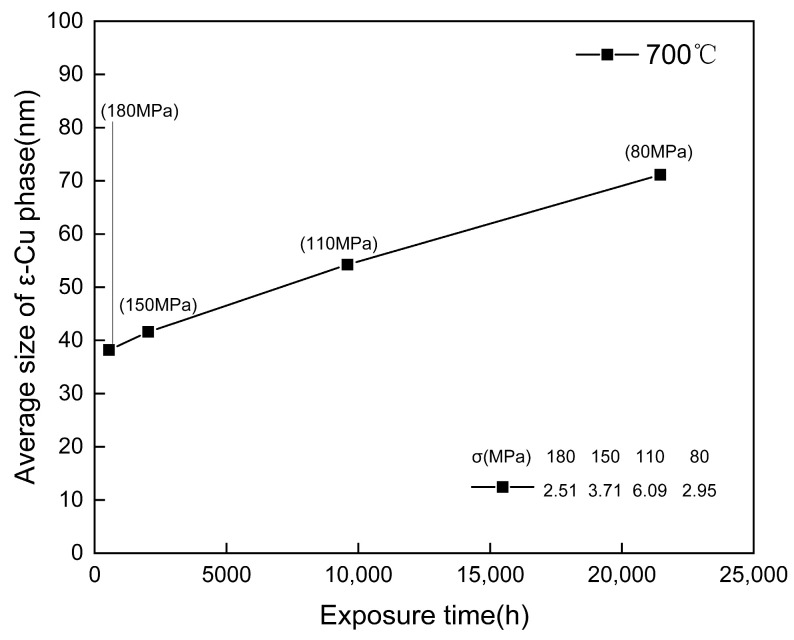
Size change of ε-Cu phase precipitates in Super304H steel after creep at 700 °C (the σ in the table in the figure is the standard deviation).

**Figure 14:**
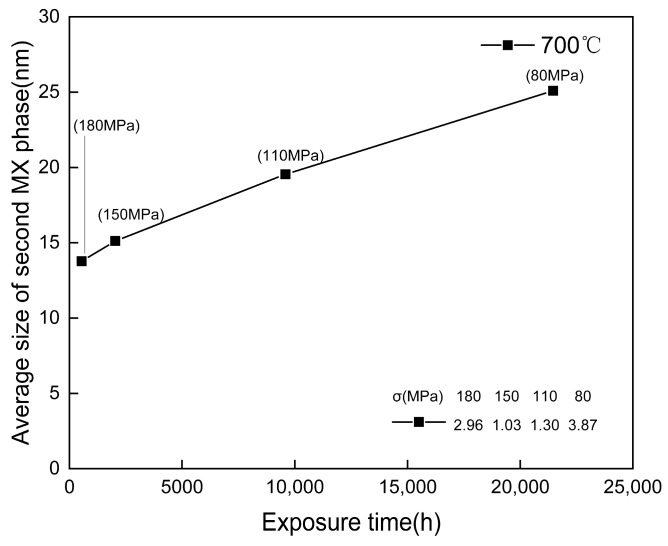
Size change of MX phase precipitates in Super304H steel after creep at 700 °C (the σ in the table in the figure is the standard deviation).

**Figure 15:**
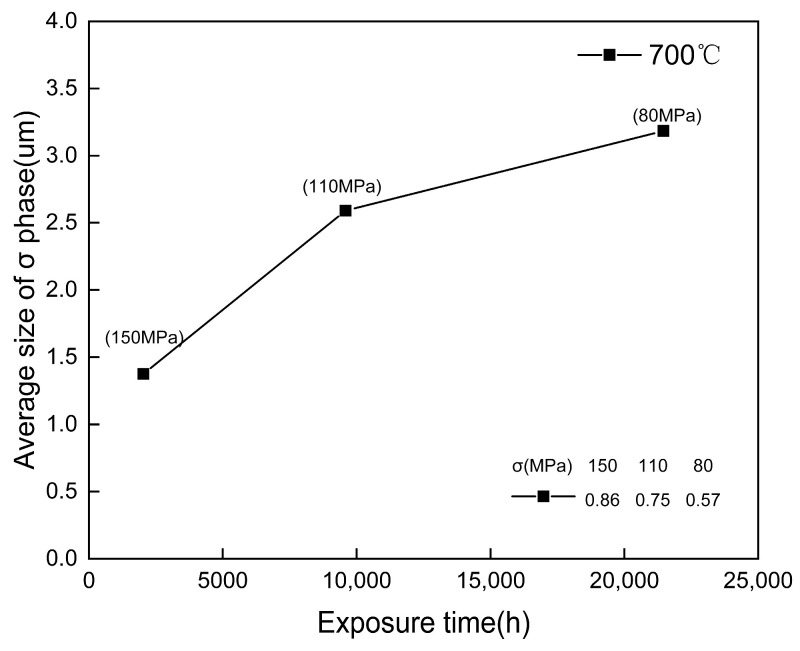
Size change of σ phase precipitates in Super304H steel after creep at 700 °C (the σ in the table in the figure is the standard deviation).

**Figure 16:**
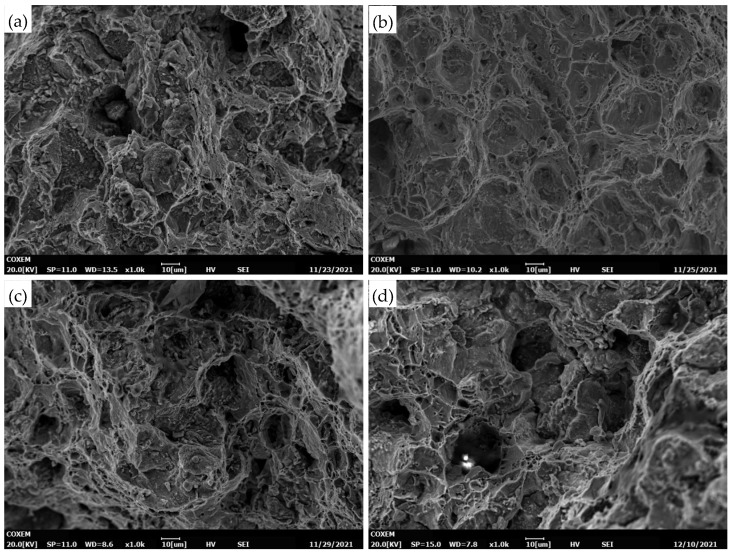
SEM images of fracture after creep rupture at 700 °C. (**a**) 180 MPa/548 h; (**b**) 150 MPa/2034 h; (**c**) 110 MPa/9584 h; (**d**) 180 MPa/21,462 h.

**Figure 17:**
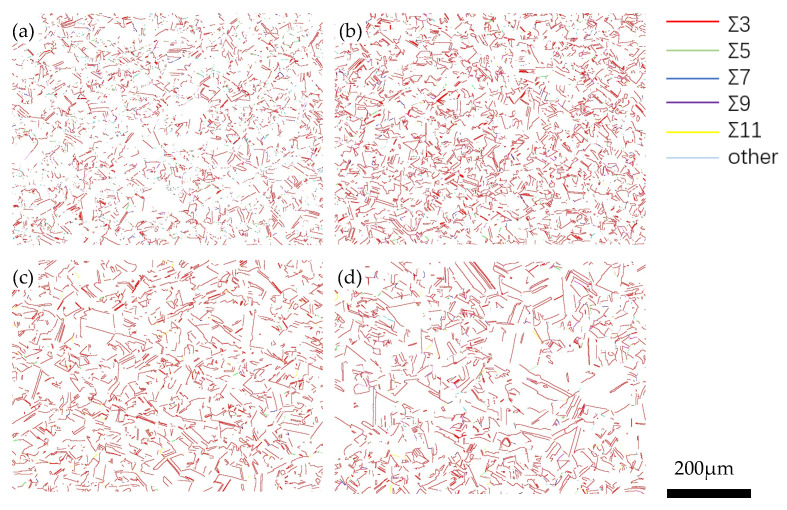
The CSL grain boundary distribution of Super304H steel under different creep stress conditions at 700 °C. (**a**) 180 MPa; (**b**) 150 MPa; (**c**) 110 MPa; (**d**) 80 MPa.

**Figure 18:**
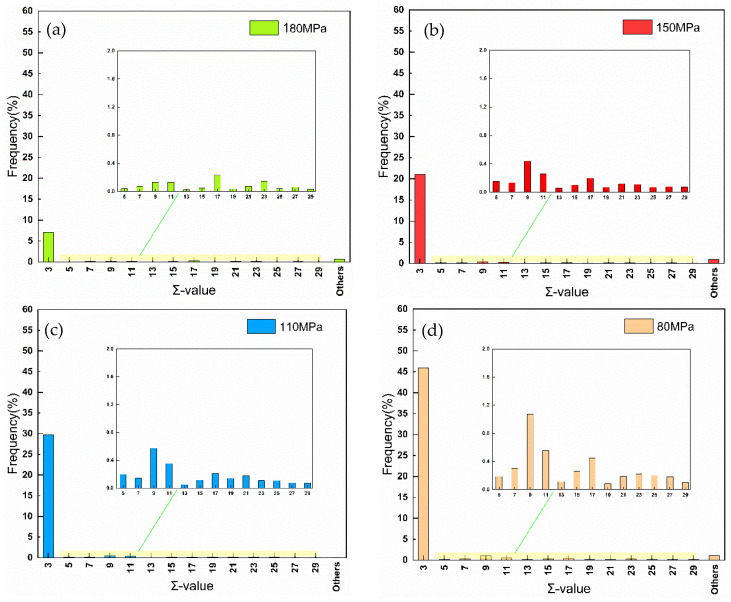
700 °C, CSL grain boundary proportion of Super304 H steel under different creep stress conditions (**a**) 180 MPa; (**b**) 150 MPa; (**c**) 110 MPa; (**d**) 80 MPa.

After 548 h of creep, the precipitation of NbCrN nitride, defined as the Z phase in the literature, is observed in the structure of Super304H steel (Figure 12). Very fine and dispersed precipitation of the ε-Cu phase was observed in austenite grains (Figure 10). After creep under different stresses, the microstructure was examined by the SEM and TEM, and the average equivalent diameters of the ε-Cu phase (Figure 13), M_23_C_6_ carbide (Figure 8), second MX phase (Figure 14), and σ phase (Figure 15) were measured. Figure 16 shows the fracture morphology of Super304H. At the same time, we used EBSD to characterize the grain boundary properties of the samples from another perspective (Figure 17 and Figure 18).

## 4. Discussion

### 4.1. Deterioration of the Creep Behavior

Figure 4 shows the trend of the microhardness of the gauge part of the samples with creep time at 700 °C. It is well known that the precipitation hardening effect is determined by the number and size of the precipitated phase [31,32,33]. During the initial creep stage (0~548 h), the hardness of both the grain and the grain boundary increases due to the increase in precipitation strengthening of M_23_C_6_ carbides and the Cu-rich phase. During the middle stage of creep (around 2034 h/150 MPa), hardening peaks, both in the matrix and at grain boundary, appear due to the precipitation hardening.

However, the hardness then decreases sharply, which may be due to the obvious coarsening of M_23_C_6_ carbides at the grain boundary and the over-aging effect of the Cu-rich phase that weakens precipitation strengthening. However, in the final creep stage (>9584 h/110 MPa), as creep time is prolonged, the hardness declines slowly and even stops. Considering the adverse effects of aggregation and coarsening of precipitates, the decreasing trend of hardness indicates the presence of some other form of precipitation strengthening that is tiny and dispersive carbidnitride MX. The MX phase, along with the Cu-rich phase and Z phase in the matrix, remains relatively stable even after long periods of creep, with its size remaining at the nanometer level (Figure 13, Figure 14 and Figure 15).

Considering the overall stable hardness value, it is foreseeable that the precipitation strengthening effect is relatively stable. In fact, unlike other austenitic steels, the microhardness of Super304H steel shows that hardness at the grain boundaries is generally higher than in the matrix, primarily due to the precipitation at the grain boundaries [15].

### 4.2. Microstructure Evolution

The precipitation of harmful phases seriously damages the microstructure stability of heat-resistant steels and reduces their performance, especially during long-term high-temperature use. Therefore, understanding the effect of second phase precipitation at different times on the microstructure is a prerequisite for its application [34].

Fine discontinuous grain boundary precipitates can increase the resistance to grain boundary sliding and also help to improve the creep strength of the steel. However, the effect of this delay decreases as the precipitates in the grains coarsen [17]. Generally speaking, due to surface defects and the disordered crystal structure, the diffusion rate of alloying elements at grain boundaries is faster than that within the grains. In addition, grain boundaries are regions with higher interface energy and serve as outlets of dislocations and vacancies, making them preferential sites for secondary precipitate formation or the precipitation of some other components. The morphology of these second phases greatly influences the overall performance of the steel, leading to embrittlement in particular [35].

It is clear that the size of M_23_C_6_ precipitates under creep load significantly increases with time. This indicates that the application of stress at a high temperature accelerates the nucleation and growth of precipitated phases at grain boundaries [36]. In addition, it is generally believed that under external stress, the interface between the coarse precipitates and the matrix may be the preferential site for microcrack nucleation. These precipitates along the interface are prone to peeling from the matrix, compromising the steel’s resistance to intergranular corrosion and fracture [37].

Figure 8 shows the size change of the M_23_C_6_ precipitates in Super304H steel after creep at 700 °C. However, no typical creep cavities were found in any of the creep samples. According to the size change of M_23_C_6_, it can be considered that the grain boundary precipitates in the creep state are metastable. The energy at the grain boundary is high, and the combination of the precipitates at the grain boundary and the matrix is still similar.

Because M_23_C_6_ carbides are inconsistent with the matrix and the diffusion of chromium in austenite is slow, the precipitation of M_23_C_6_ carbides is also slow. With the extension of creep time, their number and size gradually increase. The triangular grain boundary is particularly favorable for energy and changes the distribution of iron and chromium atoms, making it easier for M_23_C_6_ to precipitate near the σ phase [15]. During the use of Super304H steel, the precipitation process of the σ phase is an important factor affecting the functional properties of the tested steel. The increase in chromium content and strong carbide-forming elements (such as niobium and titanium) in the steel is beneficial to the precipitation of the σ phase and its appearance [38].

In general, the σ phase is considered harmful as an intermetallic compound because it is characterized by high hardness and brittleness [39]. In addition, its precipitation in austenite-based steels reduces the plasticity and ductility of the steel. The morphology of the intercrystalline phase strongly affects the overall mechanical properties. In particular, it can lead to strong embrittlement.

This detrimental effect is also reflected in the microhardness of Super304H steel. When the stress load is very high, the material retains good toughness, so the fracture morphology of the sample is a transgranular fracture. Under the influence of significant stress, the grains are torn at a faster rate, resulting in a protruding tip on the side of the sample fracture. After long-term creep, the grain boundary precipitates increase and become thicker, weakening the grain boundary and making the grain boundary near the precipitates more susceptible to tearing and integranular fracture. The fracture is mainly a transgranular fracture [4].

The fracture morphology of the sample (Figure 16) is a transcrystalline mixed fracture with elements of visible ductility, characterized by large and deep plastic deformation dimples, with some precipitated phases distributed at the bottom of the dimples. With the extension of aging time, the size and depth of the dimples decrease, and more intergranular fracture elements can be observed. However, the characteristics of a ductile fracture can still be observed after creep for 21,462 h.

The formation mechanism of the Z phase in austenitic steel is similar to that of steel with a ferrite matrix of 9~12% Cr [40]. However, unlike in martensitic steels, Z phase precipitates in austenitic steels have a positive effect on their functional properties under high-temperature and stress conditions, as they contribute to the softening of high-chromium steels. The precipitation of the Z phase occurs in austenitic steels with high niobium and nitrogen contents [41].

It is observed in Figure 12 that the Z phase is mainly precipitated inside the grains of Super304H steel. Compared to other types of heat-resistant steel, the precipitation of the Z phase plays a strengthening role at high temperatures. Sawada et al. [42] inferred that modified Z phase precipitated after 1049 h at 700 °C and after 2501 h at 650 °C, while Dae-Bum Park et al. [43] observed the precipitation of modified Z phase in the sample at 973 K and 200 MPa (t_r_ = 413 h) and believed that fine Z phases could play an important role in prolonging creep rupture failure until they grew into large size, most of these studies are about the Z phase containing vanadium in Super304H. For the Z phase, although the number of Z phase precipitates is lower compared to other precipitated phases of Super304H, under high-temperature conditions, the nano-scale fine Z phase can also provide a certain degree of strengthening.

Two types of MX precipitates with cubic structure were observed in the microstructure of Super304H austenitic steel. One of the types of larger carbides observed in the as-received Super304H austenitic steel is primary carbides. The second type of MX precipitates is nanosized fine dispersed secondary precipitates, which are mainly precipitated inside the grains. These are also among the most favorable secondary phases for stabilizing mechanical properties [44].

The precipitation process of Super304H steel was studied and analyzed by scanning electron microscopy and transmission electron microscopy. It is possible to determine the order of secondary phase precipitation in Super304H at 700 °C: primary MX phase → primary MX phase + M_23_C_6_ phase + Cu-rich phase + secondary MX phase + Z phase → primary MX phase + M_23_C_6_ phase + Cu-rich phase + secondary MX phase + Z phase + σ phase.

### 4.3. Grain Boundary Damage Assessment

Figure 17 presents the Coincidence Site Lattice (CSL) grain boundary distribution map of Super304 H steel under different creep stress conditions at 700 °C. The proportion of CSL grain boundaries among all grain boundaries under different creep stress conditions is shown in Figure 18. In the Super304H steel sample with 180 MPa creep stress, the proportion of the low-ƩCSL grain boundary can reach nearly 50%. Low-CSL grain boundaries are CSL grain boundaries of ≤29. Compared to random grain boundaries (including general high-angle grain boundaries and high-CSL grain boundaries), low-CSL grain boundaries have lower grain boundary free energy and have excellent grain boundary segregation resistance, creep resistance and intergranular corrosion resistance.

In the low-CSL grain boundaries, the Ʃ3n (n = 1, 2 and 3 or Ʃ3, Ʃ9 and Ʃ27) CSL grain boundaries occupy the main part. Figure 18 shows that except for Ʃ3, Ʃ9 and Ʃ27, they have more grain boundaries than the other low-CSL grain boundaries, which is particularly evident in long-term creep samples, and among them, the Ʃ3CSL grain boundaries are the twin boundaries accounting for the largest proportion of the low-ƩCSL grain boundaries, and their orientation difference relationship with the austenite parent grain is 60°. Among the samples subjected to creep stresses of 180 MPa, 150 MPa, 110 MPa and 80 MPa, the highest proportion of low ƩCSL grain boundaries is found in the sample subjected to creep at 80 MPa. With the increase in creep stress, the proportion of low-ƩCSL grain boundaries decreases. During the process of large strain deformation, the initial state of the Ʃ3n CSL grain boundaries has a tendency to exhibit random grain boundaries. Therefore, in creep processes involving relatively high stress, Ʃ3n grain boundaries are more likely to lose coherence with the matrix and transform into random grain boundaries. Consequently, this results in a percentage increase in random grain boundaries and a decrease in the proportion of the special low-ƩCSL grain boundaries. Therefore, it can be concluded that under conditions of high stress over short durations, grain boundary damage of Super304H steel mainly arises via the change in the grain boundary property. Conversely, under conditions of low stress over long periods of time, damage to grain boundaries is predominantly caused by harmful second phases at the grain boundary.

## 5. Conclusions

The creep behavior and microstructure evolution of Super304H (19Cr-9Ni-Nb-N) austenitic steel at different stress levels under 700 °C were systematically investigated, and the following conclusions were drawn.

In the initial creep stage, the precipitation of the second phase particles at the grain boundary and inside the grains can improve the creep resistance of the steel. With the increase in creep time, the number and size of the second phase increased and the effect of precipitation strengthening decreased. Due to the higher interfacial energy and higher diffusion rate of atoms at grain boundaries, the nucleation and growth rate of discontinuously distributed M_23_C_6_ precipitates at grain boundaries are higher than those in the matrix.

The Cu-rich phase provides an effective dispersion strengthening effect to improve creep strength. At the same time, the mechanical properties and creep resistance of the steel are improved, and this strengthening effect surpasses that of most traditional heat-resistant steels. Additionally, long-term creep precipitated a large number of nano-scale MX precipitates. Therefore, it is identified that at elevated temperatures and long periods of creep, the primary strengthening phases in Super304H steel are the Cu-rich phase and fine MX precipitates.

Precipitates of NbCrN nitrides were observed in the short-term creep samples. However, the fewer Z phases formed in Super304H offer limited benefit under high-temperature and high-stress conditions.

The second phase precipitation characteristics in Super304H subjected to creep at 700 °C varied with the stress level. Rapid σ phase precipitation at grain boundaries was observed under high stress. This weakens the grain boundaries and severely compromises the high temperature-resistant properties of Super304H steel. Finally, the precipitation sequence of Super304H steel at 700 °C was determined to be primary MX phase → primary MX phase + M_23_C_6_ phase + Cu-rich phase + secondary MX phase + Z phase → primary MX phase + M_23_C_6_ phase + Cu-rich phase + secondary MX phase + Z phase + σ phase.

Grain boundary damage of Super304H steel mainly occurs via the change in the grain boundary angle under high-stress creep, while grain boundary damage under low stress is mainly caused by the harmful second phase (mainly the σ phase) at the grain boundary.

## Figures and Tables

**Table 1:** Chemical composition of Super304H steel (mass percent, %).

	C	Si	Mn	P	S	Cu	Ni	Nb	N	Cr
As-received	0.08	0.21	0.82	0.02	0.001	3.20	9.45	0.51	0.11	18.8
ASTM A213M	0.07–0.13	Max 0.30	Max 1.00	Max 0.040	Max 0.010	2.50~ 3.50	7.5~ 10.5	0.30~ 0.60	0.05~ 0.12	17.0~ 19.0

**Table 2:** Creep rupture test data of Super304H heat-resistant steel.

Sample	Temperature (°C)	Stress (MPa)	Creep Rupture (h)	Breaking Elongation (%)	Percentage Reduction in Area (%)
1 2 3 4	700	180 150 110 80	548 2034 9584 21,462	24 17.9 15.2 7.2	36 31.9 23.5 14.2

## Data Availability

The data presented in this study are available on request from the corresponding author. The data are not publicly available due to privacy or ethical restrictions.
